# Transactions of the American Medical Association

**Published:** 1877-03

**Authors:** 


THE TRANSACTIONS OF THE AMERICAN MEDICAL ASSOCIATION: Vol. xxvii. 1876.

This is a neat volume of 715 pages, well gotten up. In it are contained Dr. J. Marion Sims' address; a plan of organization for a National American Association, and many very valuable and instructive papers, as contributions of various sections.

The code of medical ethics, as at present of force, togethor with a catalogue of the Officers of the Association, closes the volume.

